# Knowledge, risk assessment, practices, self‐efficacy, attitudes, and behaviour's towards venous thromboembolism among nurses: A systematic review

**DOI:** 10.1002/nop2.1914

**Published:** 2023-06-30

**Authors:** Khalid Al‐Mugheed, Nurhan Bayraktar

**Affiliations:** ^1^ Adult Health Nursing, College of Nursing Riyadh Elm University Riyadh Saudi Arabia; ^2^ Nursing Department, School of Health Sciences AtÄ±lÄ±m University Golbasi, Ankara Turkey

**Keywords:** attitudes, behaviours, knowledge, practices, risk assessment, self‐efficacy, venous thromboembolism

## Abstract

**Aim:**

This study reviewed the literature on nurses' knowledge, risk assessment practices, self‐efficacy, attitudes, and behaviours towards venous thromboembolism (VTE).

**Design:**

A systematic review following PRISMA guidelines.

**Methods:**

CINAHL (via EBSCO), MEDLINE (via PubMed), and Web of Science were electronic databases used to find studies published from 2010 to November 2020 in English language. A Hoy critical appraisal checklist was used to assess the risk of bias and methodologic quality.

**Results:**

This study included fourteen studies conducted on 8628 Registered Nurses. Nine of the fourteen studies examined nurses' general knowledge level regarding VTE, and five showed that most nurses had a good knowledge of VTE. Of the 14 studies, six assessed nurses' risk assessment knowledge regarding VTE, and three showed that nurses had low knowledge of VTE risk assessment. Eleven studies assessed nurses' practices concerning VTE prophylaxis; 5 of the 11 studies reported that nurses had poor and unsatisfactory levels of VTE practice. Three of the 14 studies showed that nurses had low self‐efficacy and varied beliefs. The most frequent recommendations were to create continuous educational programs and in‐service training programs (*n* = 11), followed by creating institutional protocols standardizing VTE (*n* = 6).

**Conclusions:**

Comprehensive educational programs and campaigns based on well‐established and standardized tools should be provided to nurses to improve their VTE knowledge.

## 1 INTRODUCTION

Thromboembolic diseases have become a primary cause of death, responsible for 1 in 4 deaths globally (Wendelboe & Raskob, 2016). Venous thromboembolism (VTE) is one of these diseases and includes deep vein thrombosis (DVT) and pulmonary embolism (PE; Centers for Disease Control and Prevention, 2016; Di Nisio et al., 2016). VTE has been acknowledged as the main complication among medical and surgical patients and is also known as the ‘silent killer’ of hospitalized patients (CDC, 2016).

The population‐based estimates for thrombotic conditions are limited in many countries, especially those categorized as developing (Wendelboe & Raskob, 2016). A global study was conducted to explain the epidemiology of thromboembolic diseases, which reported that the total incidence of these diseases has decreased in developed countries but is still rising in developing countries (Wendelboe & Raskob, 2016). In developed countries, VTE still significantly contributes to increased mortality and morbidity. For example, estimates suggest 60,000–100,000 Americans die from thromboembolic conditions (CDC, 2016). In developing countries in Asia, the situation is not reassuring. A study has shown that, despite the common belief that VTE is less common in Asian countries than in Western countries, the incidence rate of DVT in Asia was between 3% and 28% (Gerotziafas et al., 2018).

Venous thromboembolism can cause life‐threatening complications, prolonged hospitalization, and increased care costs (Dawoud et al., 2018; Lovely et al., 2020). According to the Centers for Disease Control (CDC), DVT increases the possibility of post‐thrombotic syndrome and PE, which affect an estimated 50% of DVT cases (CDC, 2016). In the United States, the monthly cost of treatment for DVT is an estimated $700 to $1400, and non‐pharmacological prophylaxis is estimated to cost $465 to $875 per patient (Dawoud et al., 2018).

Although VTE is a potentially life‐threatening condition, it is preventable (Khalafallah et al., 2016; Xu et al., 2018). Several organizations have developed VTE prevention guidelines to decrease VTE mortality and enhance prevention. The primary pharmacological and non‐pharmacological preventive practices recommended in these guidelines are graduated compression stockings, intermittent pneumatic compression, and anticoagulation therapy (CDC, 2016; National Institute for Health and Care Excellence, 2018). Recently, the Centers for Disease Control and Prevention has developed three main strategies to promote VTE: strengthening monitoring, best practices, and increased education about VTE (CDC, 2016).

Nurses have essential roles in preventive measures (Al‐Mugheed, Bani‐Issa, Rababa, et al., 2022; Al‐Mugheed, Bayraktar, Al‐Bsheish, et al., 2022; Al‐Mugheed, Bayraktar, Nashwan, et al., 2022). Appropriate prophylaxis is the more effective way to decrease prolonged hospitalizations, medical costs, and VTE (Al‐Mugheed & Bayraktar, 2018; Xu et al., 2018). A Canadian study showed that nurses were the most suitable healthcare providers to assess DVT prophylaxis daily (Lloyd et al., 2012). Another study showed that DVT's morbidity and mortality rates decreased after nurse's educated patients (Lavall & Costello, 2015). Implementing quality nursing care is vital to drive improvements in patients' clinical outcomes, nursing practice changes, and patient safety (Al‐Mugheed & Bayraktar, 2020; Al‐Mugheed, Bani‐Issa, Rababa, et al., 2022; Al‐Mugheed, Bayraktar, Al‐Bsheish, et al., 2022; Al‐Mugheed, Bayraktar, Nashwan, et al., 2022).

Inadequate knowledge and practice are the causes most associated with increased VTE prevalence worldwide (Silva et al., 2020). Several studies revealed inadequate knowledge of deep vein thrombosis risks and poor practices concerning the prevention of deep vein thrombosis (Ahmed et al., 2020; Al‐Mugheed & Bayraktar, 2018). Studies have also indicated that low self‐efficacy and behaviours contribute to improving the quality of VTE patient care (Silva et al., 2020; Yan et al., 2020). Increasing the knowledge and improving the practices on VTE risks and prevention to avoid complications are necessary; they may also help improve awareness and prevent this essential public health problem. However, this is the first systematic review examining relevant studies of nurses' knowledge, risk assessment, practices, self‐efficacy, attitudes, and behaviours towards VTE prophylaxis. This review may help establish appropriate nursing education and management strategies for VTE prevention and management.

## 2 METHODS

### 2.1 Eligibility criteria

The literature search for this systematic review was adopted based on the Preferred Reporting Items for Systematic Reviews and Meta‐Analyses checklist (PRISMA) guidelines. The inclusion criteria were:

- Studies using the cross‐sectional design, randomized controlled trial or quasi‐experimental study design, and pre‐post‐test design;
- Studies examining knowledge, risk assessment, practices, self‐efficacy, attitudes, and behaviours towards VTE prophylaxis;
- Studies conducted on nurses; and
- Studies using self‐reports or observation methods for data collection.

The exclusion criteria from this review were the following:

- Studies that combined nurses and other health care providers;
- Studies published as a short report or review studies;
- Studies with low quality, such as conference proceedings, dissertations, and theses; and
- Studies non English language.

### 2.2 Search strategy

Two independent researchers executed literature searches based on the consultation of a specialist health sciences librarian in advanced literature search techniques. CINAHL (via EBSCO), MEDLINE (via PubMed), and Web of Science were electronic databases used to find the studies published from 2010 to November 2020. The Web of Science search strategy was initially adapted to match other identified electronic databases. Keywords listed in Table 1 were combined with Boolean operators, including AND, OR.

**TABLE 1:** Study characteristics.

Study authors (year)	Outcome measures	Country	Study characteristics: Participants, target population, sampling method, method of data collection	Study design	Instrumentation: Type of tool, number of items, reliability
Ahmed et al. (2020)	Knowledge Practice	Egypt	Participants: 30 Target population: RN Sampling method: convenience Method of data collection: self‐completion and direct or indirect observation	Descriptive	Tool type: Researcher‐made Number of items: 16 Reliability: NA
Al‐Mugheed and Bayraktar (2018)	Knowledge Risk assessment Practice	North Cyprus	Participants: 165 Target population: RN Sampling method: NA Method of data collection: self‐completion	Descriptive, cross sectional	Tool type: researcher‐made Number of items: 47 Reliability: NA
Amira et al. (2018)	Knowledge Practice Attitudes	Egypt	Participants: 91 Target population: RN Sampling method: convenient Method of data collection: self‐reported and observations	Descriptive exploratory	Tool type: researcher‐made Number of items: 72 Reliability: 0.82
Antony et al. (2016)	Knowledge Practice	India	Participants: 100 Target population: RN Sampling method: convenient Method of data collection: self‐reported	Non experimental descriptive design	Tool type: researcher‐made Number of items: 41 Reliability: 0.8
Elder et al. (2016)	Practice	USA	Participants: 248 Target population: RN Sampling method: convenient Method of data collection: direct observations	Mixed methods	Tool type: researcher‐made Number of items: 12 Reliability: NA
Gaston and White (2013)	Risk assessment Compliance	Australia	Participants: 24 Target population: RN Sampling method: convenient Method of data collection: self‐reported and pre and post‐test	Descriptive, pre‐ and post‐test	Tool type: standardized Number of items: NA Reliability: NA
Lau et al. (2017)	Practice	USA	Participants: 24 Target population: RN Sampling method: randomized Method of data collection: pre and post‐test	Double‐blinded, cluster randomized trial	Tool type: Researcher‐made Number of items: NA Reliability: NA
Lee et al. (2014)	Knowledge Practices	USA	Participants: 221 Target Population: RN Sampling method: convenience Data collection: self‐reported	Exploratory descriptive	Tool type: standardized Number of items: 20 Reliability: 0.84
Ma et al. (2018)	Knowledge Risk assessment	China	Participants: 5097 Target population: RN Sampling method: convenience Data collection: self‐reported	Descriptive	Tool type: standardized Number of items: 68 Reliability: 0.95
Oh et al. (2016)	Knowledge Risk assessment Practice	South Korea	Participants: 452 Target population: RN Sampling method: convenience Data collection: self‐reported	A cross‐sectional descriptive	Tool type: standardized Number of items: 55 Reliability: NA
Silva et al. (2020)	Knowledge Risk assessment Self‐efficacy	Brazil	Participants: 81 Target population: RN Sampling method: convenience Data collection: self‐perceived	Cross‐sectional, descriptive	Tool type: standardized Number of items: 20 Reliability: NA
Songwathana et al. (2011)	Practice	Australia	Participants: 42 Target population: RN Sampling method: convenience Data collection: self‐reported	Research and development design	Tool type: researcher‐made Number of items: 30 Reliability: 0.90
Walker et al. (2010)	Compliance	Australia	Participants: 23 Target population: RN Sampling method: randomized Data collection: pre‐test, post‐test	A pre‐test, post‐test	Tool type: standardized Number of items: NA Reliability: NA
Yan et al. (2020)	Knowledge Attitudes Behaviours	China	Participants: 1121 Target population: RN Sampling method: convenience Data collection: self‐designed	Descriptive	Tool type: researcher‐made Number of items: 47 Reliability: NA

Abbreviations: NA, not available; RN, Registered Nurse.

### 2.3 Selection of studies and data extraction

Two independent researchers inspected abstracts and titles; then, the full text was reviewed regarding eligibility criteria. If the text matched, it was coded as ‘include’. Disagreements regarding the study's inclusion were resolved between study authors by consensus. Studies with appropriate data were included in the systematic review. The required data included study characteristics (year of publication, data collection method, participants, and sampling method), level of knowledge, risk assessment, attitudes, behaviours, self‐efficacy, practice, and recommendations of VTE.

### 2.4 Quality assessment and abstraction

The Hoy critical appraisal checklist assessed the risk of bias and methodologic quality. The main reasons to use it were that it is an easily applied tool based on an exhaustive literature review and items that showed high interrater agreement (Al‐Mugheed, Bani‐Issa, Rababa, et al., 2022; Al‐Mugheed, Bayraktar, Al‐Bsheish, et al., 2022; Al‐Mugheed, Bayraktar, Nashwan, et al., 2022; Hoy et al., 2012). The Hoy critical appraisal checklist includes a 10‐item checklist with two domains: External validity (target sample, frame of the sample, sampling method, and nonresponse bias minimal) and internal validity (data collected, case definition, validity and reliability of study instrument, and data collection mode). Two assessors assessed each paper for bias risk, and discussions resolved any discrepancies.

## 3 RESULTS

### 3.1 Study characteristics

**FIGURE 1:**
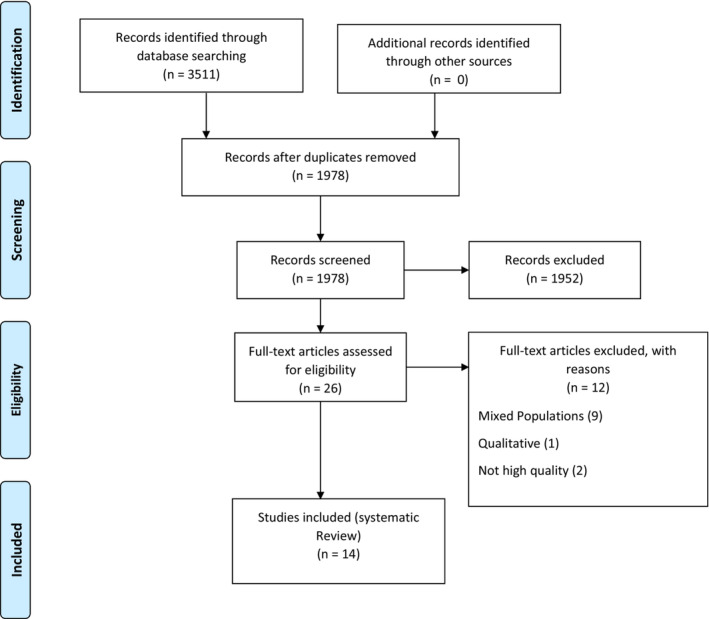
PRISMA flow diagram.

A total of 3511 articles were identified from the initial search in four different electronic databases. After the deletion of duplicates, 1978 studies were addressed for further screened. After reading the abstract and full texts, 1952 articles were excluded. Full‐text articles assessed for eligibility included 26 articles. Twelve studies were excluded for the following reasons: mixed populations (9), qualitative (1), and not high quality (2). Fourteen studies were included in the final review (Figure 1). Fourteen studies conducted on 8628 Registered Nurses were included in this study. Most studies had been performed in developed countries (*n* = 10; Elder et al., 2016; Gaston & White, 2013; Lau et al., 2017; Lee et al., 2014; Ma et al., 2018; Oh et al., 2016; Silva et al., 2020; Songwathana et al., 2011; Walker et al., 2010; Yan et al., 2020). Seven studies used self‐reports (Amira et al., 2018; Antony et al., 2016; Gaston & White, 2013; Lee et al., 2014; Ma et al., 2018; Oh et al., 2016; Songwathana et al., 2011). Most studies used convenience sampling and a cross‐sectional, descriptive design, see Table 1.

### 3.2 Nurses' general knowledge of VTE

Except for five studies (Amira et al., 2018; Elder et al., 2016; Gaston & White, 2013; Songwathana et al., 2011; Walker et al., 2010), all others examined nurses' general knowledge level regarding VTE. The level of knowledge in most studies was classified as good, high, average, and satisfactory (Al‐Mugheed & Bayraktar, 2018; Antony et al., 2016; Lee et al., 2014; Ma et al., 2018; Silva et al., 2020). The remaining studies characterized the level of knowledge as unsatisfactory, not optimistic, and fair (Ahmed et al., 2020; Amira et al., 2018; Oh et al., 2016; Yan et al., 2020). The overall results showed that most nurses had good knowledge levels regarding VTE. In Al‐Mugheed and Bayraktar's (2018) study, most nurses had numerous correct answers regarding the definition and causes of VTE. In one study, half of the nurses answered questions correctly regarding the initial diagnostic test of VTE (Silva et al., 2020). In Antony et al. (2016), nurses correctly answered items regarding VTE's pathophysiology, signs, and symptoms. In contrast, Oh et al. (2016) found that nurses had low correct answering rates regarding VTE diagnosis; only 15% correctly knew a CT scan as the initial diagnostic test for PE, see Table 2.

**TABLE 2:** Knowledge, risk assessment, practices, self‐efficacy, attitudes and behaviour's towards venous thromboembolism among nurses.

Study authors (year)	Knowledge level	Self‐ efficacy and beliefs	Risk assessment knowledge	Practice	Attitudes	Behaviour's
Ahmed et al. (2020)	Unsatisfactory	—	—	Unsatisfactory	—	—
Al‐Mugheed and Bayraktar (2018)	High	—	Inadequate	Poor	—	—
Amira et al. (2018)	Unsatisfactory	—	—	Unsatisfactory	Satisfactory	—
Antony et al. (2016)	Average	—	—	Poor	—	—
Elder et al. (2016)	—	Varied	—	—	Varied	—
Gaston and White (2013)	—	—	High after education	Increased after education	—	—
Lau et al. (2017)	—	—	—	Increased after education	—	—
Lee et al. (2014)	Good	—	Poor	Moderate	—	—
Ma et al. (2018)	Satisfactory	—	Satisfactory	Poor	—	—
Oh et al. (2016)	Fair		Low	Moderate	—	—
Silva et al. (2020)	Good	Low	Good	—	—	—
Songwathana et al. (2011)	—	—	—	High	—	—
Walker et al. (2010)	—	—	—	Increased after education	—	—
Yan et al. (2020)	Not Optimistic	—	—	—	Positive	Low

### 3.3 Nurses' knowledge of risk assessment VTE

Of the 14 studies, six assessed nurses' risk assessment knowledge regarding VTE (Al‐Mugheed & Bayraktar, 2018; Gaston & White, 2013; Lee et al., 2014; Ma et al., 2018; Oh et al., 2016; Silva et al., 2020). The risk assessment level was classified as high, good, inadequate, low, and poor. In half of these studies (*n* = 3), nurses had low knowledge regarding VTE risk assessment (Al‐Mugheed & Bayraktar, 2018; Lee et al., 2014; Oh et al., 2016). One study reported high risk assessment knowledge after attending to education (Gaston & White, 2013). Specific items demonstrating low risk assessment knowledge were identified; more than half of nurses had low correct answers regarding hormone replacement therapy, surgery, and cancer as risk factors for VTE (Al‐Mugheed & Bayraktar, 2018; Oh et al., 2016). In another study, nurses correctly answered <50% of questions regarding VTE risk factors, including inflammatory bowel disease (27.83%), obesity (47.05%), stillbirth or miscarriage of more than 3 (45.11%), and oral contraceptives (33.88%; Ma et al., 2018), see Table 2.

### 3.4 Nurses' practices level on VTE prophylaxis

Of the 14 studies, 11 assessed nurses' practices concerning VTE prophylaxis (Ahmed et al., 2020; Al‐Mugheed & Bayraktar, 2018; Amira et al., 2018; Antony et al., 2016; Gaston & White, 2013; Lau et al., 2017; Lee et al., 2014; Ma et al., 2018; Oh et al., 2016; Songwathana et al., 2011; Walker et al., 2010). The practice levels of the nurses on VTE prophylaxis were categorized as poor, unsatisfactory, and moderate. Of the 11 studies, five reported nurses had poor VTE practice levels (Ahmed et al., 2020; Al‐Mugheed & Bayraktar, 2018; Amira et al., 2018; Antony et al., 2016; Ma et al., 2018). Three studies reported that nurses had VTE practice levels after education (Gaston & White, 2013; Lau et al., 2017; Walker et al., 2010). Only one study reported a high VTE prophylaxis practice level (Songwathana et al., 2011). Two studies reported a moderate level of VTE prophylaxis (Lee et al., 2014; Oh et al., 2016). Most nurses reported poor practices regarding VTE mechanical prophylaxis devices in different studies. For example, Silva et al. (2020) found that only a quarter of nurses used VTE mechanical prophylaxis among patients. One study reported that half of the nurses had poor practice with mechanical devices for VTE prevention (Lee et al., 2014). Al‐Mugheed and Bayraktar (2018) found that most nurses had poor practices related to VTE mechanical prophylaxis devices. In contrast, encouraging early mobilization and leg exercise were frequently implemented practices of most nurses in different studies (Ma et al., 2018; Oh et al., 2016; Silva et al., 2020; Songwathana et al., 2011), see Table 2.

### 3.5 Self‐efficacy, beliefs, attitudes, and behaviours of the nurses towards VTE prevention

Only three of the 14 studies considered the nurses' self‐efficacy, attitudes, and behaviours towards VTE prevention (Elder et al., 2016; Silva et al., 2020; Yan et al., 2020); these studies showed that nurses had low self‐efficacy and varied beliefs. Two studies were conducted among nurses concerning attitudes and behaviours towards VTE; one reported positive attitudes and a low level of behaviours towards VTE prevention (Yan et al., 2020). However, most nurses had a high behaviour rate related to regularly assessing the VTE risks of those hospitalized and providing VTE prevention education for patients (Yan et al., 2020). The nurses showed varied attitudes in using their clinical decision‐making to offer and administer low‐molecular‐weight heparin doses to the patient (Elder et al., 2016), see Table 2.

### 3.6 Recommendations for improving nurses' knowledge, self‐efficacy, beliefs, attitudes, behaviours, and practices for VTE

Table 3 shows recommendations from identified literature regarding improving nurses' knowledge, risk assessment, attitude, behaviour, and practice towards VTE. The most frequent recommendations in most studies were continuous educational programs and in‐service training programs (*n* = 11; Ahmed et al., 2020; Al‐Mugheed & Bayraktar, 2018; Amira et al., 2018; Antony et al., 2016; Gaston & White, 2013; Lau et al., 2017; Ma et al., 2018; Oh et al., 2016; Silva et al., 2020; Walker et al., 2010; Yan et al., 2020), followed by the creation of institutional protocols standardizing VTE (*n* = 6; Al‐Mugheed & Bayraktar's, 2018; Elder et al., 2016; Lee et al., 2014; Oh et al., 2016; Silva et al., 2020; Songwathana et al., 2011), and continuous evaluation VTE care systems in hospitals (*n* = 4; Gaston & White, 2013; Lee et al., 2014; Oh et al., 2016; Yan et al., 2020).

**TABLE 3:** Recommendations for improving nurses' knowledge, risk assessment, practices, self‐efficacy, attitudes, and behaviour's towards venous thromboembolism among.

	Ahmed et al. (2020)	Al‐Mugheed and Bayraktar (2018)	Amira et al. (2018)	Antony et al. (2016)	Elder et al. (2016)	Gaston and White (2013)	Lau et al. (2017)	Lee et al. (2014)	Ma et al. (2018)	Oh et al. (2016)	Silva et al. (2020)	Songwathana et al. (2011)	Walker et al. (2010)	Yan et al. (2020)
Creation of institutional protocols standardizing VTE		√			√			√		√	√	√		
Continuous educational programs and in‐service training programs	√	√	√	√		√	√		√	√	√		√	√
Integrated up‐to‐date knowledge and practice regarding VTE prevention	√	√												
Providing resources and materials for implementing basics VTE guidelines prevention									√					√
Daily observations to supervise suitable implementation of VTE guidelines prevention				√					√					
Adding educational courses associated with VTE in academic nursing curricula				√										
Continuous evaluation VTE care systems in hospitals						√		√		√				√
Identify mechanisms to address barriers to VTE assessment and prevention								√				√		√
Proper feedback to improve attitude & behaviour's associated with controlling VTE					√	√								√

### 3.7 Risk of bias

In general, most studies reported having a high‐quality level with low bias. The most commonly found ‘Definitely High’ and ‘Probably High’ were related to confounding and performance. Of the 14 studies, two reported ‘Definitely High’ in terms of confounding, and two reported ‘Probably High’ related to performance, see Table 4 for further details.

**TABLE 4:** Risk of bias.

	Study title	Selection	Confounding	Performance	Attrition/Exclusion	Detection	Selective reporting
Ahmed et al. (2020)	Assessment of nurses' knowledge and practice about venous thrombo embolism for cancer surgery patients	PL	PL	DL	DL	DL	DL
Al‐Mugheed and Bayraktar (2018)	Knowledge and practices of nurses on deep vein thrombosis risks and prophylaxis: A descriptive cross sectional study	PL	DL	DL	DL	DL	PL
Amira et al. (2018)	Nurses' performance regarding venous thromboembolism prophylaxis at intensive care unit	DL	PL	DL	DL	PL	DL
Antony et al. (2016)	Assessment of knowledge and self‐reported clinical practice on prevention of deep vein thrombosis (DVT) among staff nurses	DL	DL	DL	DL	DL	DL
Elder et al. (2016)	Hidden barriers to delivery of pharmacological venous thromboembolism prophylaxis: The role of nursing beliefs and practices	PH	PH	DH	DL	PL	PL
Gaston and White (2013)	Venous thromboembolism (VTE) risk assessment: Rural nurses' knowledge and use in a rural acute care hospital	DL	DL	DL	DL	DL	DL
Lau et al. (2017)	Effectiveness of two distinct web‐based education tools for bedside nurses on medication administration practice for venous thromboembolism prevention: A randomized clinical trial	PL	DL	PL	DL	DL	PL
Lee et al. (2014)	Evaluation of hospital nurses' perceived knowledge and practices of venous thromboembolism assessment and prevention	DL	DH	PH	PH	PL	PL
Ma et al. (2018)	Nurses' objective knowledge regarding venous thromboembolism prophylaxis: A national survey study	PL	DL	DL	PL	PL	DL
Oh et al. (2016)	Clinical nurses' knowledge and practice of venous thromboembolism risk assessment and prevention in South Korea: a cross‐sectional survey	DL	DL	PH	DL	DL	PL
Silva et al. (2020)	Nurses' knowledge, risk assessment, and self‐efficacy regarding venous thromboembolism	DL	DL	DL	DL	PL	PL
Songwathana et al. (2011)	Evaluation of a clinical nursing practice guideline for preventing deep vein thrombosis in critically ill trauma patients	DL	DL	DL	PL	PL	PL
Walker et al. (2010)	Testing the effect of a targeted intervention on nurses' compliance with “best practice” mechanical venous thromboembolism prevention	PL	PL	DL	DL	DL	DL
Yan et al. (2020)	Nurses' knowledge, attitudes, and behaviours towards venous thromboembolism prophylaxis: How to do better	DL	DH	PL	PL	PL	PL

Abbreviations: DH, definitely high; DL, definitely low; PH, probably high; PL, probably low.

## 4 DISCUSSION

Venous thromboembolism prevention is the most potent way to positively impact patient outcomes and decrease extended hospitalizations, as VTE incidence has increased over time among hospitalized patients (Yan et al., 2020). This is the first systematic review to examine nurses' knowledge, risk assessment, practices, self‐efficacy, attitudes, and behaviours towards VTE. This research included four studies conducted on 8628 Registered Nurses from eight countries.

The overall results showed that most nurses had good knowledge of VTE, compared with other studies reporting that nurses had inadequate knowledge of VTE (Fangfei & Juliane, 2010; Zhou et al., 2019). The results showed that nurses had adequate knowledge of the causes, pathophysiology, signs, and symptoms of VTE. This finding does not align with a recent study that found medical staff had poorly scored regarding the causes and pathology of VTE (Zhou et al., 2019). The possible reasons may be a result of methodological variations by using different questionnaires in the studies and a lack of nursing staff participation.

The present study results indicate that most participants had inadequate and poor knowledge of VTE risk assessment. Other studies also showed poor knowledge of VTE risk assessment among other healthcare providers (Kesieme et al., 2016; Shah et al., 2020; Zobeiri & Najafi, 2011). This poor knowledge of VTE risk assessment could indicate that nurses thought VTE risk assessment was not among their daily tasks. However, little literature has determined that nurses are fully aware of their role in monitoring VTE risk assessment (Ma et al., 2018).

According to American College of Chest Physicians (ACCP) guidelines, the risk of VTE is classified as low, moderate, high, and very high risk (Kearon et al., 2012). In the identified literature, nurses showed poor knowledge regarding high and very high‐risk items of VTE risk assessment, such as hormone replacement therapy, surgery, obesity, inflammatory bowel disease, and cancer (Al‐Mugheed & Bayraktar’, 2018; Ma et al., 2018; Oh et al., 2016). Conversely, Korubo et al. (2015) found that most participants had correctly answered regarding risk factors and clinical presentation of VTE. However, nurses must perform a routine daily assessment for patients at risk of VTE.

Nurses can play a crucial role in VTE prevention by applying their knowledge to provide suitable prophylactic measures and assessing VTE risk for VTE patients (Al‐Mugheed & Bayraktar, 2018). Therefore, nurses must exhibit best practices regarding VTE guidelines (Al‐Mugheed & Bayraktar, 2021a, 2021b). The identified literature does not reflect this view (Ahmed et al., 2020; Al‐Mugheed & Bayraktar, 2018; Amira et al., 2018; Antony et al., 2016; Ma et al., 2018). The results reported that nurses had a poor level of VTE practices. This finding does not align with studies conducted among medical staff that showed good practice towards VTE prophylaxis (Ebrahimpur et al., 2016; Zobeiri & Najafi, 2011). Poor practices may result from poor financial rewards, supervision, and a lack of training programs and standardized protocols (Rababa et al., 2022). Although the overall practice level was poor, most nurses in the identified literature frequently implemented practices like encouraging early mobilization and leg exercise. A possible explanation may be that this type of practice does require additional resources or effort, or they learned from their experience (Al‐Mugheed, Bani‐Issa, Rababa, et al., 2022; Al‐Mugheed, Bayraktar, Al‐Bsheish, et al., 2022; Al‐Mugheed, Bayraktar, Nashwan, et al., 2022).

Regarding the attitudes, behaviours, self‐efficacy, and beliefs towards VTE prevention VTE, the results indicated that the nurses had low and varied perceptions. A possible explanation may be that nurses lacked confidence when implementing VTE prophylaxis due to a lack of knowledge and resources. Also, nurses believed that some VTE prophylaxis measures were not their primary daily duty (Lee et al., 2014; Silva et al., 2020). Positive behaviour improvement requires leaders to frequently evaluate nurses' behaviours and attitudes regarding VTE prophylactic intervention and allocate the time to prepare them via education, supervise new staff, and appoint a VTE clinical nurse consultant (Li et al., 2020).

The most recommended solutions were continuous educational programs to improve nurses' knowledge. The studies revealed that teaching session interventions significantly improved practice and risk assessment (Gaston & White, 2013; Lau et al., 2017; Walker et al., 2010). Continuing VTE education programs play a significant role in VTE prevention, and departmental managers should expand efforts for nurses' training (Gaston & White, 2013).

Venous thromboembolism prophylaxis protocols are recommended to improve the practices of healthcare providers regarding VTE prevention (Li et al., 2020). The National Institute for Health and Care Excellence recently endorsed the VTE prophylaxis protocol as one of the 10 most crucial patient safety practices (NICE, 2018). Creating institutional protocols standardizing VTE was the second most frequent recommendation in the studies. Using protocols standardizing VTE showed significant improvement in VTE patient outcomes and saved lives by preventing complications (Amira et al., 2018; Collins & MacLellan, 2010).

### 4.1 Implications for nursing practice

This review provides nurse managers and policymakers with evidence on enhancing nurses' preparation for VTE prevention in their units. Prevention of VTE decreases mortality rates in critical care patients, shortens hospital stays, and minimizes related costs. A need exists for qualified VTE nurses with proper education. During the COVID‐19 pandemic, the need for education of nurses about VTE prevention is critical as the region has witnessed an increased number of patients admitted to critical care units. The overwhelming needs for qualified VTE nurse still represent a significant barrier to the quality of care in a clinical unit. On‐the‐job training and continuous education programs are essential to prepare qualified VTE nurses.

### 4.2 Limitations

The present study used the PRISMA checklist to consider all possible VTE dimensions and recommendations from identified studies. Most studies were of good quality. Among the limitations were that most studies used descriptive design, few studies were conducted in developing countries, and the studies were conducted in only eight countries.

## 5 CONCLUSION

This is the first systematic review to examine nurses' knowledge, risk assessment, practice, self‐efficacy, attitudes, and behaviours towards VTE. Across the identified studies, the nurses' knowledge level regarding VTE was higher than in risk assessment and practice, thus highlighting the gap between theory and practice, which must be addressed systematically to enhance patient care and reduce VTE incidences.

## AUTHOR CONTRIBUTIONS

All listed authors meet the authorship criteria and that all authors are in agreement with the content of the manuscript. Have made substantial contributions to conception and design, or acquisition of data, or analysis and interpretation of data; Al‐Mugheed K, Bayraktar N. Been involved in drafting the manuscript or revising it critically for important intellectual content; Bayraktar N. Given final approval of the version to be published. Al‐Mugheed K, Bayraktar N. Agreed to be accountable for all aspects of the work in ensuring that questions related to the accuracy or integrity of any part of the work are appropriately investigated and resolved. Al‐Mugheed K, Bayraktar N.

## FUNDING INFORMATION

This research did not receive any specific grant from funding agencies in the public, commercial, or not‐for‐profit sectors.

## CONFLICT OF INTEREST STATEMENT

The authors declare no conflict of interest.

## PATIENT CONSENT

No patient or public contribution.

## Data Availability

Data available on request from the authors.
